# Multiple Drug-Induced Stress Responses Inhibit Formation of Escherichia coli Biofilms

**DOI:** 10.1128/AEM.01113-20

**Published:** 2020-10-15

**Authors:** Nataliya A. Teteneva, Sergey V. Mart’yanov, María Esteban-López, Jörg Kahnt, Timo Glatter, Alexander I. Netrusov, Vladimir K. Plakunov, Victor Sourjik

**Affiliations:** a Max Planck Institute for Terrestrial Microbiology, Marburg, Germany; b Center for Synthetic Microbiology (SYNMIKRO), Marburg, Germany; c Winogradsky Institute of Microbiology, Federal Research Center Fundamentals of Biotechnology, Russian Academy of Sciences, Moscow, Russia; d Department of Microbiology, Lomonosov Moscow State University, Moscow, Russia; University of Tokyo

**Keywords:** *Escherichia coli*, biofilms, drug repurposing, stress response

## Abstract

In most ecosystems, bacteria exist primarily as structured surface-associated biofilms that can be highly tolerant to antibiotics and thus represent an important health issue. Here, we explored drug repurposing as a strategy to identify new antibiofilm compounds, screening over 1,000 compounds from the Prestwick Chemical Library of approved drugs for specific activities that prevent biofilm formation by Escherichia coli. Most growth-inhibiting compounds, which include known antibacterial but also antiviral and other drugs, also reduced biofilm formation. However, we also identified several drugs that were biofilm inhibitory at doses where only a weak effect or no effect on planktonic growth could be observed. The activities of the most specific antibiofilm compounds were further characterized using gene expression analysis, proteomics, and microscopy. We observed that most of these drugs acted by repressing genes responsible for the production of curli, a major component of the E. coli biofilm matrix. This repression apparently occurred through the induction of several different stress responses, including DNA and cell wall damage, and homeostasis of divalent cations, demonstrating that biofilm formation can be inhibited through a variety of molecular mechanisms. One tested drug, tyloxapol, did not affect curli expression or cell growth but instead inhibited biofilm formation by suppressing bacterial attachment to the surface.

**IMPORTANCE** The prevention of bacterial biofilm formation is one of the major current challenges in microbiology. Here, by systematically screening a large number of approved drugs for their ability to suppress biofilm formation by Escherichia coli, we identified a number of prospective antibiofilm compounds. We further demonstrated different mechanisms of action for individual compounds, from induction of replicative stress to disbalance of cation homeostasis to inhibition of bacterial attachment to the surface. Our work demonstrates the potential of drug repurposing for the prevention of bacterial biofilm formation and suggests that also for other bacteria, the activity spectrum of antibiofilm compounds is likely to be broad.

## Abstract (precis)

The prevention of bacterial biofilm formation is one of the major current challenges in microbiology. Here, by systematically screening a large number of approved drugs for their ability to suppress biofilm formation by Escherichia coli, we identified a number of prospective antibiofilm compounds. We further demonstrated different mechanisms of action for individual compounds, from induction of replicative stress to disbalance of cation homeostasis to inhibition of bacterial attachment to the surface. Our work demonstrates the potential of drug repurposing for the prevention of bacterial biofilm formation and suggests that also for other bacteria, the activity spectrum of antibiofilm compounds is likely to be broad.

## INTRODUCTION

Biofilms are the most widespread form of bacterial existence in nature (1–4). Within biofilms, bacteria are typically embedded in a matrix that consists of polysaccharides, proteins, and extracellular DNA (5, 6). Biofilms are formed at various types of interfaces, including epithelial layers and surfaces of catheters, and many chronic infections are caused by bacteria that are present in biofilms (1). Since cells within biofilms have low sensitivity to many antimicrobial compounds (7), the prevention of biofilm formation is important in disease treatment. A number of different alternative approaches are currently being explored as antibiofilm strategies, including surface modification to prevent bacterial adhesion or assembly of the biofilm matrix, specific enzymes to degrade the biofilm matrix, and inhibition of bacterial signaling or quorum sensing using small molecules (8–12).

Escherichia coli is a common and medically relevant model for biofilm research (13, 14). The major matrix components of E. coli biofilms are amyloid protein fibers known as curli (15, 16). The matrix of E. coli also includes other components such as colanic acid, cellulose, and poly-β-1,6-*N*-acetyl-d-glucosamine, the content of which varies by strain and is dependent on growth conditions (17–19). E. coli biofilm formation is a highly regulated process that includes initial attachment and biofilm maturation steps and depends on a number of signaling pathways that regulate curli biosynthesis (14, 19). The expression of structural components of curli, including the major curlin CsgA, is under the control of the master regulator CsgD that is expressed dependent on the activity of the stationary-phase sigma factor σ^S^ and the interplay between several diguanylate cyclases and phosphodiesterases that control the level of the second messenger cyclic di-GMP (c-di-GMP) (20–22). CsgD, which regulates the inverse coordination between planktonic motile and biofilm sessile lifestyles, is mediated by mutual inhibition between the σ^S^/CsgD curli and flagellar gene expression control cascades (23, 24). The latter consists of three classes of flagellar genes, where the master regulator FlhDC (class I) induces the expression of class II (middle) genes, including the flagellum-specific sigma factor FliA, which in turn activates the expression of class III (late) flagellar genes (25).

In this study, we screened the Prestwick Chemical Library of over 1,000 U.S. Food and Drug Administration (FDA)-approved drugs to identify new compounds that are active against submerged biofilms of E. coli. This library was recently used to identify drugs that impact human gut bacteria (26) or inhibit the growth of several pathogenic bacteria in planktonic culture and also in biofilms (27–29). In contrast, the prime focus of our screen was to identify drugs that specifically inhibit E. coli biofilm formation while having a weak effect or no effect on planktonic growth. Indeed, we report several prospective antibiofilm compounds that were active against both laboratory and pathogenic E. coli strains at doses below the growth-inhibitory concentration. The antibiofilm effect of most of these compounds could be explained by the inhibition of curli expression and, in some cases, the activation of motility, apparently due to the induction of several different stress responses or due to the inhibition of bacterial attachment.

## RESULTS

### Identification of novel antibacterial and antibiofilm drugs.

In order to identify new antibiofilm compounds, we screened 1,280 off-patent drugs approved for human use by the FDA from the Prestwick Chemical Library for their ability to suppress the growth as well as the formation of submerged biofilms of E. coli K-12 strain W3110, a common biofilm model (19, 23). In this screen, E. coli cultures were incubated in 96-well microtiter plates at 10 μM final concentrations of individual compounds, and bacterial growth and biofilm formation were quantified (see Materials and Methods).

**FIG 1:**
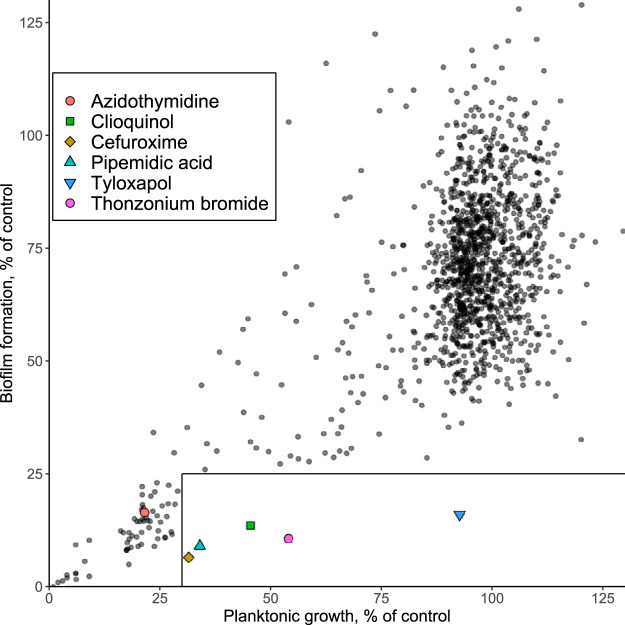
Screen for antibiofilm compounds within the Prestwick Chemical Library of FDA-approved drugs. Growth of planktonic E. coli cultures and formation of biofilms were measured in the presence of 10 μM individual library compounds. Biofilm formation was quantified using CV staining (see Materials and Methods). An untreated culture was used as a control. The boxed-off section indicates cultures in which treatment resulted in a >75% reduction of biofilm formation while having a <70% effect on planktonic growth. Compounds that were chosen for future work are indicated.

We observed that under these conditions, many compounds had detectable inhibitory effects on E. coli growth (Fig. 1 and Table 1; see also Data Set S1 in the supplemental material). This group of compounds included mostly established antibacterial drugs but, more surprisingly, also antiviral and other drugs. These novel antibacterial compounds might be promising as potential drugs, although their activity spectra and mechanisms of action require future investigation. Most of these growth-inhibitory compounds also proportionally reduced the biomass of surface-attached cells in a crystal violet (CV) assay (Fig. 1), suggesting that their effect on biofilm formation is the consequence of growth inhibition. Nevertheless, several compounds had a comparatively stronger effect on biofilm formation than on growth. The activities of all potentially specific antibiofilm drugs, which at 10 μM reduced CV staining by >75% but inhibited planktonic growth by <70%, were verified in an additional screen. This left a total of 5 prospective compounds, including 3 established antibacterial drugs, clioquinol, pipemidic acid, and cefuroxime sodium salt, but also 2 surfactants, tyloxapol and thonzonium bromide (Table 1).

**TABLE 1:** Antibacterial actions of selected compounds at a 10 μM final concentration

Compound^*a*^	Class(es)	Mechanism of action	Mean planktonic growth (% of control) ± SE	Mean biofilm formation (% of control) ± SE
TX	Mucolytic, surfactant	Reduces the surface tension of the mucus	93 ± 15	16 ± 14
TZ	Surfactant	Cationic surface-active compound	54 ± 2	11 ± 6
CQ	Antiamebic, antifungal, antiseptic	Probable chelator	37 ± 6	11 ± 2
PA	Antibacterial	Gyrase inhibitor	34 ± 7	9 ± 4
CF	Antibacterial	2nd-generation cephalosporin, inhibitor of cell wall synthesis	32 ± 4	6 ± 1
AZT	Antiretroviral	Inhibitor of reverse transcriptase	22 ± 0.5	16 ± 5

a TX, tyloxapol; TZ, thonzonium bromide; CQ, clioquinol; PA, pipemidic acid; CF, cefuroxime sodium salt; AZT, azidothymidine (zidovudine).

### Prospective compounds suppress biofilm formation by laboratory and uropathogenic E. coli strains.

**FIG 2:**
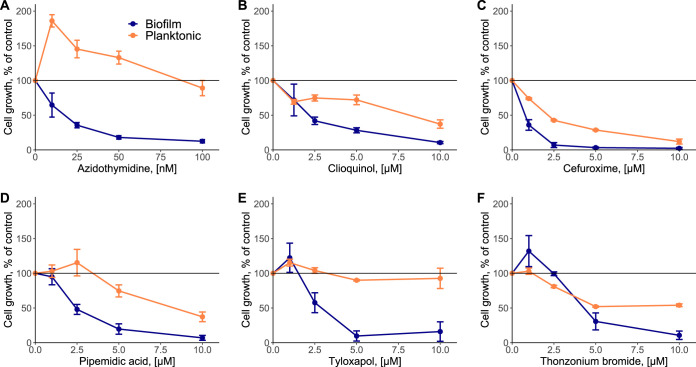
Dose-dependent effects of the indicated drugs on planktonic culture growth and biofilm formation. Growth and biofilm formation of E. coli W3110 were measured as described in Materials and Methods in the presence of various concentrations of azidothymidine (A), clioquinol (B), cefuroxime sodium salt (C), pipemidic acid (D), tyloxapol (E), and thonzonium bromide (F). All experiments were performed in triplicates. Error bars indicate standard errors.

We subsequently subjected these drugs to a more detailed analysis of their antibiofilm activities. Additionally, we included an antiviral drug, zidovudine (3′-azido-3′-deoxythymidine; azidothymidine), that showed high activity against both planktonic growth and biofilm formation in our screen (Fig. 1). All of these compounds showed dose-dependent inhibition of the biomass of surface-attached cells and, except for tyloxapol, also reduced the growth of planktonic E. coli cultures (Fig. 2). Nevertheless, the antibiofilm activity of all drugs was consistently higher than growth inhibition, which in most cases was particularly apparent at low concentrations. This turned out to also be true for azidothymidine, which already showed antibiofilm activity in the nanomolar concentration range, where it had no measurable effects on or even weakly stimulated planktonic growth.

We further confirmed the potential of these selected drugs to suppress biofilm formation by three uropathogenic E. coli (UPEC) strains, EcoR-50, EcoR-64, and DSMZ 10650. Antibiofilm activity of the tested drugs was observed for all three UPEC strains at concentrations similar to those for E. coli W3110 (Fig. S1). As a minor difference between these isolates, EcoR-50 biofilms were the most sensitive to azidothymidine, pipemidic acid, clioquinol, and thonzonium bromide, whereas cefuroxime showed the highest activity against DSMZ 10650 biofilms. In general, EcoR-64 biofilms were the least sensitive to the tested drugs, and surprisingly, biofilm formation was even stimulated by low concentrations of thonzonium bromide. These strain-specific differences in sensitivity to drugs seemingly correlate with the strains’ sensitivity to commonly used antibiotics (Table S1). Here again, EcoR-64 showed the highest levels of resistance, while EcoR-50 was the most sensitive. Nevertheless, even for EcoR-64, biofilm formation could be efficiently suppressed by several compounds, including clioquinol and tyloxapol, indicating that the identified drugs should be applicable against biofilms of antibiotic-resistant UPEC strains.

Finally, we tested the effects of these compounds on UPEC biofilm formation on urinary catheters. Here, we focused on DSMZ 10650 since in our preliminary experiments, this strain showed the most pronounced biofilm formation on catheters when growing in donor urine. Several compounds showed inhibitory effects under these conditions, most notably tyloxapol and cefuroxime (Fig. S2). These effects were observed both at 30°C, a temperature that is commonly used to study E. coli biofilm formation, as well as at body temperature, 37°C.

### Most antibiofilm compounds inhibit curli and activate flagellar expression.

**FIG 3:**
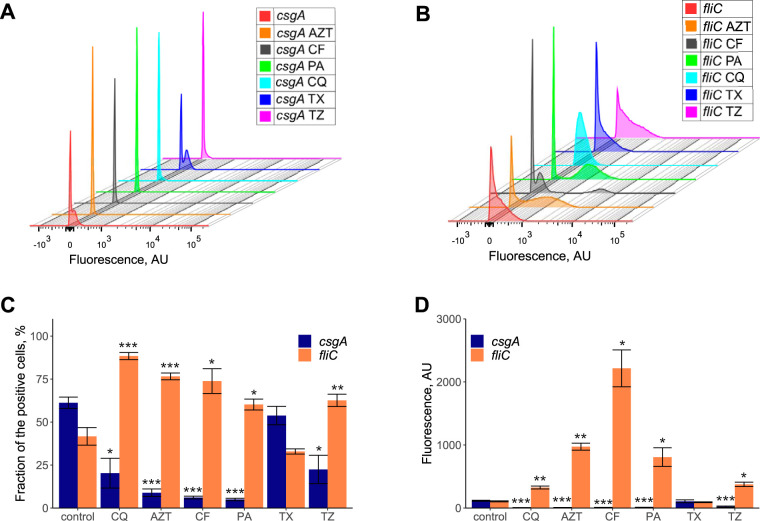
Effects of the indicated drugs on the expression of curli and flagellar genes. (A and B) Representative measurements of the activities of the *csgA* (A) and *fliC* (B) promoter reporters using flow cytometry (see Materials and Methods) in populations of E. coli W3110 cells grown in tryptone broth (TB) medium or in TB medium supplemented with 0.1 μM azidothymidine (AZT), 2.5 μM cefuroxime sodium salt (CF), 10 μM pipemidic acid (PA), 1 μM clioquinol (CQ), 2.5 μM tyloxapol (TX), or 5 μM thonzonium bromide (TZ). The *y* axis in panels A and B represents the cell count, with a total of 50,000 events for each experiment. (C and D) Corresponding fractions of fluorescent cells (C) and median fluorescence (D). All experiments were performed in triplicates. Error bars indicate standard errors. *P* values were calculated using a Mann-Whitney test (*, *P < *0.05; **, *P < *0.01; ***, *P < *0.001). AU, arbitrary units.

To elucidate the possible modes of action of the identified antibiofilm compounds, we first assayed their effects on curli genes that encode the major matrix component of E. coli. We used a green fluorescent protein (GFP) reporter of the *csgA* promoter that controls the expression of the main curli operon and determined reporter activity in cells that were recovered from the surface of the incubation well (see Materials and Methods). As reported previously (23), the *csgA* promoter showed bimodal expression in a population of untreated W3110 bacteria, with fractions of *csgA*-positive and -negative cells (Fig. 3A). Since curli genes in E. coli are known to be counterregulated with the flagellar regulon (23, 24), we also monitored the activity of the flagellin (*fliC*) promoter. Under our conditions, the activity of the *fliC* promoter was also seemingly bimodal (Fig. 3B). We observed that upon incubation with all antibiofilm drugs except tyloxapol, both the fraction of *csgA*-positive cells and the overall reporter activity decreased dramatically (Fig. 3A, C, and D). Consistently, these compounds also enhanced the activity of the *fliC* promoter, unmasking its bimodality and strongly increasing the fluorescence of positive cells (Fig. 3B to D). Thus, clioquinol, pipemidic acid, cefuroxime, thonzonium bromide, and azidothymidine all inhibit the expression of the curli matrix while also activating motility and therefore cell dispersion, which provides a likely mechanism of the antibiofilm activity of these compounds.

### Tyloxapol prevents attachment of E. coli to plastic surfaces.

**FIG 4:**
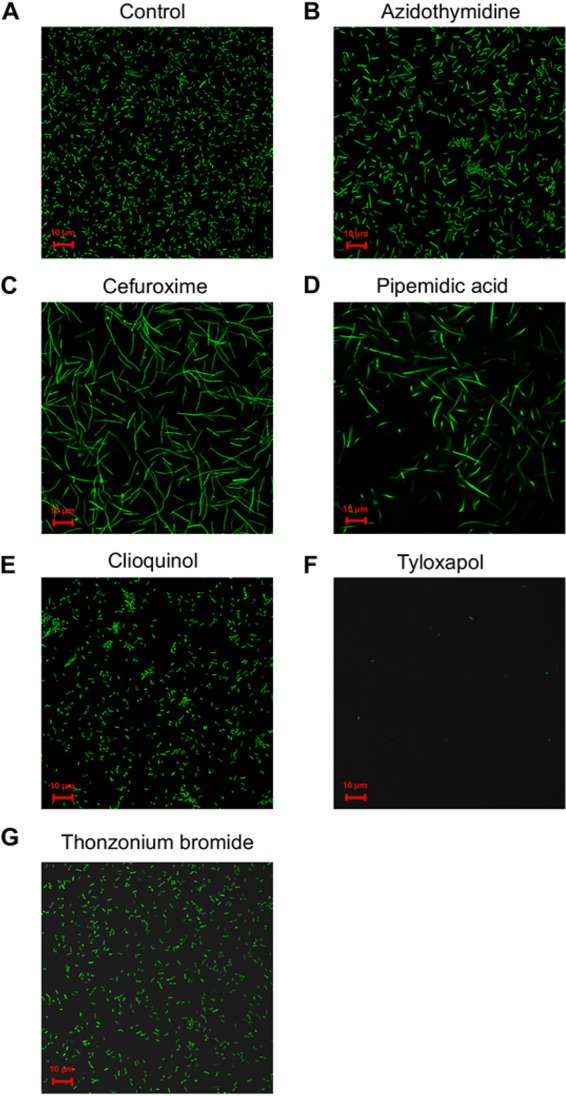
Attachment of E. coli cells to a plastic surface under treatment. Shown are representative images of cultures grown in microscopy plates (see Materials and Methods) for 6 h in the absence of treatment (A) or in the presence of azidothymidine (B), cefuroxime sodium salt (C), pipemidic acid (D), clioquinol (E), tyloxapol (F), and thonzonium bromide (G) at the concentrations indicated in Fig. 3. Attached cells were washed and imaged in PBS.

In contrast to other drugs, tyloxapol showed no significant effect on curli or flagellar expression (Fig. 3), suggesting that it suppresses E. coli W3110 biofilms by a mechanism different from the inhibition of matrix biosynthesis. Since tyloxapol is a known surface-active compound (30), we investigated whether it could affect the surface attachment of E. coli. Indeed, nearly no attachment to the plastic surface of microscopy wells was observed for E. coli cells in the presence of tyloxapol (Fig. 4), whereas other drugs, including thonzonium bromide, which has also been described as a surface-active compound (31), had little effect on attachment. Nevertheless, microscopy analysis showed that even subinhibitory concentrations of pipemidic acid and cefuroxime resulted in a substantial elongation of E. coli cells. This effect might be explained by the activities of these drugs that are known to inhibit bacterial cell wall biosynthesis (cefuroxime) and gyrase (pipemidic acid) (Table 1), both of which could lead to a (partial) suppression of cell division. Interestingly, certain cell elongation was also observed upon incubation with azidothymidine, consistent with the general similarity of the effects of azidothymidine and pipemidic acid on E. coli (see below).

The observed effect of tyloxapol on attachment is apparently surface specific since tyloxapol did not prevent the attachment of E. coli W3110 to the glass surface in a commercial Bioflux chamber that was used to study biofilm formation under flow (Fig. S3). In contrast, all drugs that induced cell elongation at subinhibitory concentrations strongly suppressed the early stages of E. coli biofilm formation in this flow chamber, possibly due to the facilitated detachment of elongated daughter cells under flow. Clioquinol and thonzonium bromide had no apparent effects on these early biofilms, presumably because curli expression becomes important only at later stages of submerged biofilm formation by E. coli (14, 19).

### Antibiofilm compounds induce different stress responses in E. coli.

**FIG 5:**
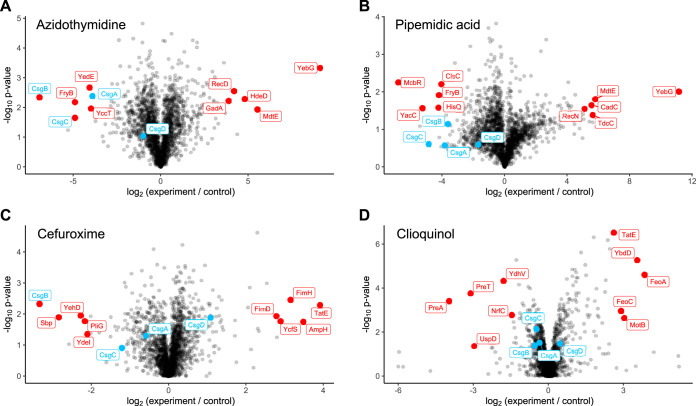
Global changes in protein levels upon treatment with the indicated drugs. Protein changes were analyzed using mass spectrometry (see Materials and Methods) for cultures grown in the presence of azidothymidine (A), pipemidic acid (B), cefuroxime sodium salt (C), and clioquinol (D) at the concentrations indicated in Fig. 3. Data are shown as volcano plots, where the *x* axis represents the fold change of the label-free quantification (LFQ) intensity of each protein, while the *y* axis represents the −log_10_ *P* value determined by Student’s *t* test. The most up- and downregulated proteins are highlighted in red; selected curli proteins are highlighted in blue.

In order to better understand the underlying mechanisms of curli inhibition by clioquinol, pipemidic acid, cefuroxime, and azidothymidine, we performed analyses of changes in the proteome composition that are induced in surface-attached cells by these drugs at concentrations that inhibit biofilm formation by approximately 50%. Although the expected reductions of the levels of structural curli proteins (CsgA, CsgB, and CsgC) were indeed observed for all compounds, many changes in protein levels were compound specific (Fig. 5, Table 2, Fig. S4, and Data Set S2). Interestingly, also, the expression of the regulator CsgD was inhibited only in some cases (pipemidic acid and azidothymidine) but not in other cases. This suggests that curli downregulation might occur through several different mechanisms, which was further confirmed by comparisons of global regulatory changes mediated by individual compounds (Fig. S5). Nevertheless, treatment with azidothymidine and pipemidic acid led to highly similar changes in the E. coli proteome, indicating that these two drugs have an at least partly shared mechanism of action. This could also be noticed at the level of the most highly up- or downregulated proteins, where both drugs appeared to induce the DNA damage SOS response (Fig. 5A and B and Table 2). This might be consistent with the established activities of these compounds that might interfere with DNA replication (Table 1). The acid stress response and the multidrug efflux pump were also activated by both compounds. Pipemidic acid (Fig. 5A and B and Table 2) and, to a lesser extent, also azidothymidine (Data Set S1) further led to reduced levels of the transcription factor McbR that was previously implicated in E. coli biofilm formation (32).

**TABLE 2:** The most up- and downregulated proteins in E. coli cultures grown in the presence of the indicated compounds^*a*^

Azidothymidine			Cefuroxime sodium salt			Pipemidic acid			Clioquinol		
Protein	Fold change	Protein description	Protein	Fold change	Protein description	Protein	Fold change	Protein description	Protein	Fold change	Protein description
YebG	9.11	DNA damage-inducible protein YebG	TatE	3.92	Sec-independent protein translocase protein TatE	YebG	11.15	DNA damage-inducible protein YebG	FeoA	3.84	Ferrous iron transport protein A
MdtE	5.53	Multidrug resistance protein MdtE	AmpH	3.48	d-Alanyl-d-alanine-carboxypeptidase/endopeptidase AmpH	MdtE	5.80	Multidrug resistance protein MdtE	YbdD	3.55	Uncharacterized protein YbdD
HdeD	4.81	Acid resistance membrane protein HdeD	FimH	3.15	Protein FimH	TdcC	5.63	Threonine/serine transporter TdcC	MotB	3.03	Motility protein B
FrlB	4.37	Fructoselysine 6-phosphate deglycase	YcfS	2.89	Probable l,d-transpeptidase YcfS	TdcD	5.63	Propionate kinase	FeoC	2.90	Ferrous iron transport protein C
RecD	4.19	RecBCD enzyme subunit RecD	DnaT	2.80	Primosomal protein 1	CadC	5.56	Transcriptional activator CadC	TatE	2.61	Sec-independent protein translocase protein TatE
GadA	3.89	Glutamate decarboxylase alpha	FimD	2.79	Outer membrane usher protein FimD	FrlB	5.42	Fructoselysine 6-phosphate deglycase	FecB	2.45	Fe^3+^ dicitrate-binding periplasmic protein
DinI	3.74	DNA damage-inducible protein I	FtsL	2.77	Cell division protein FtsL	RecN	5.12	DNA repair protein RecN	PlaP	2.42	Low-affinity putrescine importer PlaP
TdcC	3.70	Threonine/serine transporter TdcC	FtsI	2.54	Peptidoglycan synthase FtsI	CroE	5.06	Putative lambdoid prophage e14 transcriptional regulatory protein	FecA	2.36	Fe^3+^ dicitrate transport protein FecA
RmuC	3.61	DNA recombination protein RmuC	YgaC	2.45	pH stress-inducible protein YgaC	DctR	5.04	HTH-type transcriptional regulator DctR	FecE	2.29	Fe^3+^ dicitrate transport ATP-binding protein FecE
PaaZ	3.28	Oxepin-CoA hydrolase/3-oxo-5,6-dehydrosuberyl-CoA semialdehyde dehydrogenase PaaZ	FtsB	2.29	Cell division protein FtsB	FdhF	5.01	Formate dehydrogenase H	GabP	2.25	GABA permease
DgcJ	−3.34	Putative diguanylate cyclase YeaJ	ShiA	2.09	Shikimate transporter	CsgA	−3.83	Major curlin subunit	TomB	−1.30	Hha toxicity modulator TomB
PhoA	−3.58	Alkaline phosphatase PhoA	CspD	2.05	Cold shock-like protein CspD	ClsC	−4.05	Cardiolipin synthase C	HybO	−1.37	Hydrogenase 2 small chain
HisQ	−3.74	Histidine transport system permease protein HisQ	PliG	−2.17	Inhibitor of g-type lysozyme	YibI	−4.06	Uncharacterized protein YibI	PliG	−1.38	Inhibitor of g-type lysozyme
CsgA	−3.90	Major curlin subunit	YehD	−2.28	Uncharacterized fimbria-like protein YehD	HolB	−4.19	DNA polymerase III subunit delta′	HybA	−1.40	Hydrogenase 2 operon protein HybA
YccT	−3.98	Uncharacterized protein YccT	Sbp	−2.84	Sulfate-binding protein	FryB	−4.20	Fructose-like phosphotransferase enzyme IIB component 1	NrfC	−1.46	Protein NrfC
YedE	−4.06	Inner membrane protein YedE	CsgB	−3.34	Minor curlin subunit	HisQ	−4.24	Histidine transport system permease protein HisQ	YdhV	−1.79	Uncharacterized oxidoreductase YdhV
CsgC	−4.90	Curli assembly protein CsgC				CsgC	−4.86	Curli assembly protein CsgC	UspD	−2.96	Universal stress protein D
FryB	−4.90	Fructose-like phosphotransferase enzyme IIB component 1				YacC	−5.26	Putative lipoprotein YacC	PreT	−3.11	NAD-dependent dihydropyrimidine dehydrogenase subunit PreT
NlpC	−5.30	Probable endopeptidase NlpC				YbiO	−5.43	Moderate-conductance mechanosensitive channel YbiO	PreA	−3.97	NAD-dependent dihydropyrimidine dehydrogenase subunit PreA
CsgB	−6.93	Minor curlin subunit				McbR	−6.81	HTH-type transcriptional regulator McbR			

a Proteins related to curli assembly and regulation are shaded. HTH, helix-turn-helix.

In contrast, profiles of expression changes induced by cefuroxime or clioquinol had little overlap with those of other compounds and showed only weak correlations between each other. E. coli treated with cefuroxime showed elevated expression levels of several proteins involved in cell wall synthesis and division (Table 2), which is consistent with its function as a cell wall-inhibiting drug (Table 1). Cefuroxime also activated the expression of fimbriae (FimH, FimG, and FimD) and the twin-arginine transporter component TatE. Clioquinol induced TatE, along with the induced expression of the FeoC uptake system for ferrous iron and the ZntA export system for zinc and cadmium (Table 2), which might be consistent with its role as metal ion chelator (33).

### The antibiofilm effect of clioquinol is related to the homeostasis of divalent cations.

**FIG 6:**
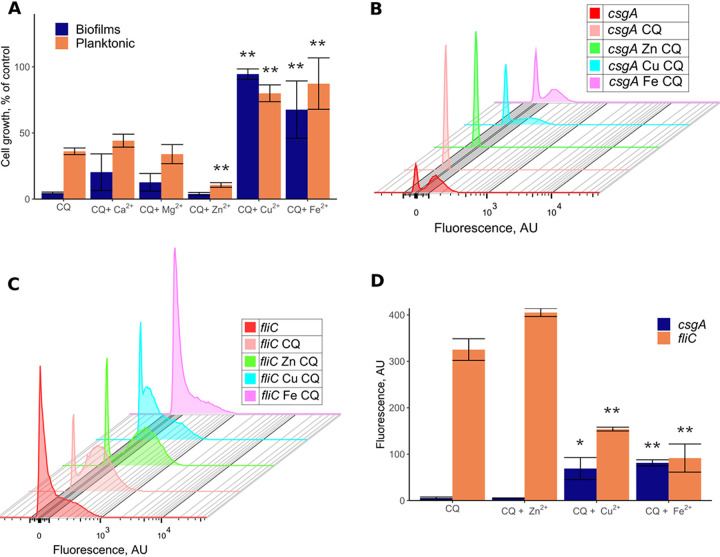
Effects of divalent cations on growth, biofilm formation, and gene expression in clioquinol-treated E. coli cultures. (A) Changes in cell growth and biofilm formation in the presence of clioquinol (CQ) alone or the combination of clioquinol (CF) and the indicated divalent cations (10 μM ZnCl_2_, 10 μM CuSO_4_, or 10 μM FeSO_4_). All values are normalized to the values for untreated cultures. (B to D) Activities of the *csgA* (B) and *fliC* (C) promoter reporters and corresponding changes in median fluorescence (D) in cultures with or without treatment with 10 μM clioquinol and divalent cations, as indicated. The *y* axis in panels B and C represents the cell count, with a total of 50,000 events for each experiment. All experiments were performed in triplicates. Representative measurements are shown in panels B and C. Error bars in panels A and D indicate standard errors. *, *P* < 0.05; **, *P* < 0.01.

In order to investigate whether the observed antibiofilm activity of clioquinol is indeed related to the homeostasis of divalent cations, we investigated how this effect is influenced by the addition of divalent cations. Indeed, copper and ferrous iron cations restored the ability of E. coli W3110 to form biofilms in the presence of clioquinol and also relieved the growth of planktonic cultures in a dose-dependent manner (Fig. 6A and Fig. S6), largely eliminating the effect of clioquinol at an equimolar concentration (10 μM). In contrast, the addition of zinc suppressed the growth of planktonic cultures even further, whereas the addition of magnesium or calcium cations did not change the growth of planktonic cultures or biofilm formation. Consistently, copper and iron relieved the clioquinol-mediated repression of curli genes, whereas zinc further enhanced their repression (Fig. 6B and D and Fig. S7A). An opposite pattern was observed for flagellar gene expression (Fig. 6C and D and Fig. S7B). Notably, none of the tested cations by themselves had any effects on gene expression or biofilm formation.

## DISCUSSION

Most studies dedicated to discovering new antibiofilm drugs are aimed at the specific targeting of biofilm-related pathways (8–10). While this hypothesis-guided approach is potentially promising, it is limited by our poor understanding of the complexity of regulation that underlies the transition to the biofilm state. As an alternative strategy, several recent studies relied on systematic large-scale screening of drugs for their biocidal activity against planktonic cell cultures, with subsequent retesting of the identified biocidal compounds for their antibiofilm activity (28, 29). This approach enables the identification of biocidal compounds that are similarly active against planktonic and biofilm cultures, but it is unlikely to yield antibiofilm compounds that do not suppress planktonic growth.

The aim of this study, in contrast, was to identify novel drugs that act specifically against biofilms of E. coli among a library of the FDA-approved drugs by designing the screen to focus on drugs that show stronger biofilm suppression than inhibition of growth. This enabled us to identify several antibiofilm compounds, among which three are established antibacterial biocides (clioquinol, pipemidic acid, and cefuroxime), one is an antiviral drug (azidothymidine), and two are surfactants, tyloxapol and thonzonium bromide (although the latter is also a known antiseptic). Out of the identified drugs, only cefuroxime has been previously shown to be active against bacterial biofilms (34, 35). Notably, although the screen was performed for the laboratory strain W3110 of E. coli that is commonly used as a model for biofilm formation (19, 23), most of these compounds similarly suppressed the biofilms of several tested UPEC strains, and several of them affected UPEC biofilms formed on catheters. This indicates the potential clinical applicability of these drugs, although more extensive testing on clinical isolates of E. coli would be necessary to verify it. Furthermore, such a repurposing strategy is likely to yield novel antibiofilm drugs for other bacterial species as well.

Besides identifying these prospective compounds, we could at least partly characterize the mechanisms of their antibiofilm activity. For one of the identified compounds, the nonionic surfactant tyloxapol, this activity is apparently based on the direct suppression of surface attachment since even at high concentrations, tyloxapol had no effect on bacterial growth or the expression of curli genes. Tyloxapol is commonly used as a mucolytic agent for the treatment of pulmonary diseases, and it might also possess anti-inflammatory activity (36), but to our knowledge, its antibiofilm (or antibacterial) activity has not been reported so far. Although its spectrum of action remains to be tested, it likely conditions surfaces to prevent the attachment of type I fimbriae and/or flagella, the major adhesins of E. coli W3110 under our experimental conditions (23).

The mode of action of all other compounds, including clioquinol, pipemidic acid, cefuroxime, azidothymidine, and thonzonium bromide, is apparently related to the suppression of the expression of curli, the major component of the E. coli biofilm matrix. It might be further enhanced by stimulation of the expression of the motility-related genes. This results in a combination of reduced aggregation and enhanced dispersion of bacteria. This finding is overall consistent with the importance of curli for E. coli biofilm formation (15, 16) and with the known counterregulation between curli and motility genes (23, 24). Since curli are important for biofilm formation in not only laboratory but also pathogenic E. coli strains (14, 37), the suppression of curli expression could explain why these drugs are active against UPEC biofilms.

Interestingly, however, our analysis of global changes in protein levels suggested that mechanisms that resulted in curli gene inhibition were different between individual drugs. Two of the drugs, pipemidic acid and azidothymidine, induced similar changes in protein expression, apparently related to the DNA damage response. This is consistent with the established mode of action of pipemidic acid, a known inhibitor of bacterial topoisomerase II (gyrase) (38). Although azidothymidine (zidovudine) is known as an inhibitor of viral reverse transcriptase (39), it can apparently similarly interfere with E. coli DNA replication and/or repair. Notably, the antibacterial activity of azidothymidine has been reported previously, although its mechanism of action remained unknown (40). The exact pathway that links the observed induction of DNA damage stress to the repression of curli remains to be elucidated, but in both cases, we observed reduced levels of the transcription factor McbR, a positive regulator of E. coli biofilm formation (32). The apparent weak growth-activating effect of low concentrations of azidothymidine also remains to be understood.

Cefuroxime belongs to the cephalosporin group of antibiotics that inhibits cell wall synthesis (41), and consistently, we observed that it caused cell elongation and induced the expression of several cell wall proteins along with the expression of type I fimbriae. Despite these elevated levels of adhesins, the formation of biofilm was strongly reduced through the repression of curli. Interestingly, cefuroxime may also be applicable as an antibiofilm agent against biofilms of the Gram-positive bacterium Staphylococcus aureus (35).

Finally, clioquinol (5-chloro-7-iodoquinolin-8-ol) affected the expression of several proteins involved in iron and zinc transport. Clioquinol is an established chelator of zinc, copper, and iron, and it is also known to act as an ionophore (33). These properties might explain the multiple effects of clioquinol on animal cells (33, 42, 43) as well as its reported antibacterial (44), antifungal (45), and antiprotozoal (46) properties. The same chelator/ionophore activity that perturbs the homeostasis of divalent cations is the likely cause of the clioquinol-mediated suppression of curli expression and biofilm formation by E. coli since the effect of clioquinol could be suppressed by the addition of equimolar amounts of copper or ferrous (II) iron. Interestingly, however, the addition of zinc rather potentiated the effect of clioquinol, although zinc itself had no effect on biofilm formation. The addition of iron was previously shown to stimulate curli expression and biofilm formation by uropathogenic E. coli by inducing oxidative stress (47), so iron chelation by clioquinol might cause biofilm inhibition. This might also explain the cumulative effects of clioquinol and zinc since the addition of zinc might perturb iron uptake (48, 49). Additionally, zinc is known to affect cellular levels of c-di-GMP by inhibiting the diguanylate cyclase DgcZ (50).

In summary, by performing an extensive screen, we could identify several drugs that specifically suppress biofilm formation by both commensal and pathogenic E. coli strains while having a weak effect or no effect on bacterial growth at a given concentration. While one of the identified antibiofilm drugs specifically prevented adhesion, the effect of other drugs was due to suppressed curli production and therefore cell aggregation. This apparently occurred due to the induction of different stress responses, including disrupted homeostasis of divalent cations, DNA damage, or perturbations to cell wall biosynthesis. The same treatments also induced the expression of motility genes, thus likely stimulating cell dispersion. Such stress-induced suppression of biofilm formation and induction of motility was unexpected since in E. coli, curli expression is under positive regulation whereas flagellar expression is under negative regulation by the general stress response (23, 24), and biofilms typically promote stress resistance (7). It remains to be investigated whether this effect is common and whether stress induction by these or other drugs might also inhibit biofilm formation in other bacteria. Finally, suppression of curli expression might have clinical applications besides inhibition of biofilm formation since curli fibers are known to be generally important for E. coli pathogenicity (14, 37).

## MATERIALS AND METHODS

### Strains and culture conditions.

E. coli W3110 was used here as the model for biofilm formation (19, 23). Additionally, three uropathogenic E. coli (UPEC) strains, EcoR-50, EcoR-64, and DSMZ 10650, were used for comparison. Bacteria were grown at 30°C, a temperature that favors E. coli biofilm formation (19, 23), in tryptone broth (TB) medium (10 g tryptone and 5 g NaCl per liter) supplemented with antibiotics where necessary. The same W3110 strain but with a genomic enhanced GFP (eGFP) reporter under the control of the *rplL* promoter (23) was used for microscopy. Promoter activities were measured using GFP reporter plasmids for the *csgA*, *csgD*, *fliA*, *fliC*, and *flhD* promoters (23, 51, 52).

### Biofilm growth and quantification.

Biofilms were quantified using a standard crystal violet (CV) assay on microtiter plates (53), with modifications. Briefly, cultures of E. coli W3110 grown overnight in TB in a rotary shaker at 30°C were diluted 1:100 into fresh TB medium and grown at 220 rpm to the mid-exponential phase (optical density at 600 nm [OD_600_] = 0.5) at 30°C. The culture was diluted in fresh TB medium to an OD_600_ of 0.05, and 300 μl was loaded into a 96-well plate (Corning Costar, flat bottom; Sigma-Aldrich, Germany). The OD_600_ of the planktonic culture was measured after 20 to 24 h of stationary incubation at 30°C, and the liquid culture was then removed from the wells. The wells were washed once with 1× phosphate-buffered saline (PBS) (8 g NaCl, 0.2 g KCl, 1.44 g Na_2_HPO_4_, 0.24 g KH_2_PO_4_), and the biofilms were then fixed with 300 μl of 96% ethanol. After 20 min, ethanol was removed, and the plates were left to dry under a fume hood for 40 min and then stained with 300 μl of a 0.1% crystal violet solution for 15 min. Crystal violet was removed, and biofilms were washed twice with the same buffer. The remaining CV stain in biofilms was extracted by adding 300 μl of 96% ethanol for 35 min, and the OD_595_ was measured. All the measurements were performed with an Infinite 200 Pro multimode plate reader (Tecan Group Ltd., Switzerland).

### Biofilm growth on urinary catheters.

Cultures of UPEC strains grown overnight in TB in a rotary shaker at 30°C were diluted 1:100 in fresh TB medium and grown at 220 rpm to the mid-exponential phase (OD_600_ = 0.5) at 30°C. Cultures were subsequently diluted in filter-sterilized human urine from a female donor to an OD_600_ of 0.05, and 1.5 ml of the culture was loaded into a 24-well plate (Corning Costar, flat bottom; Sigma-Aldrich, Germany) containing 1-cm pieces of a 12-Fr silicone Foley catheter (Azid Bonz, Germany) and grown for 48 h at 30°C or 37°C. For biofilm quantification, catheter pieces were taken from the wells, washed with PBS, allowed to dry on a paper towel, and stained with 1.5 ml of a 0.1% CV solution for 10 min. Next, the tubing was rinsed with distilled water using a syringe and allowed to dry. The remaining CV stain in biofilms was extracted by adding 1.5 ml of 96% ethanol for 35 min, and the OD_595_ was then measured in three technical replicates per catheter piece.

### Library screening.

The Prestwick Chemical Library (Prestwick Chemical, Illkirch-Graffenstaden, France) contains 1,280 compounds at 10 mM in dimethyl sulfoxide (DMSO). These compounds were diluted 10-fold in DMSO using an Integra Viaflo 96/384 robotic liquid-handling system (Switzerland), and 3 μl was added to the bacterial culture, resulting in a 10 μM final concentration.

### Fluorescence microscopy.

Bacterial cultures were prepared as described above. Two hundred microliters of the diluted culture was seeded per well into 96-well microscopy plates with untreated surfaces (μ-Plate 96-well black; ibidi GmbH, Germany). Bacteria were grown in TB medium at 30°C without shaking for 6 h. Where indicated, the tested compounds were added to the medium during growth. After cultivation, planktonic cells were carefully removed and replaced with 200 μl of PBS. Fluorescent cells were visualized using a Zeiss Axio Observer LSM 880 inverted laser scanning microscope equipped with a C-Apochromat 40×/1.2 Water Corr-UV-VIS-IR objective and a 514-nm argon laser.

### Microfluidics.

Microfluidic assays were performed by using a Bioflux 200 system (Fluxion Biosciences Inc., USA). Cells were grown in TB medium at 30°C until an OD_600_ of 0.5 was reached. The cells were then diluted in fresh TB until an OD_600_ of 0.05 was reached and flushed into the channels for 3 h at 0.5 dyn/cm^2^. Afterwards, cells were removed from the input well, and fresh TB medium supplemented with the respective compounds at the indicated concentrations was flushed into the channels overnight at 0.5 dyn/cm^2^. An exception was tyloxapol, where the medium already contained tyloxapol during the first 3 h of incubation. Imaging was performed on a Nikon Eclipse Ti-U fluorescence microscope equipped with an iXon3 897 electron-multiplying charge-coupled-device (EMCCD) camera using a 40× objective and a GFP (excitation, 470 ± 20 nm; emission, 525 ± 25 nm) filter set. Two positions per channel were imaged per strain. Quantification of whole fluorescence was performed using Fiji software.

### Flow cytometry.

Bacteria were grown as described above for fluorescence microscopy except that TB medium was supplemented with 50 μg/ml kanamycin to select for reporter plasmids. The planktonic culture was carefully removed, 200 μl PBS was added to the well, and the attached cells were removed from the surface by pipetting and scratching using a 1-ml pipette tip. The obtained suspension was centrifuged for 5 min at 4,500 × *g*, and the pellet was then resuspended in PBS and vortexed vigorously to disrupt all remaining cell aggregates. Samples were diluted 20-fold in PBS, and fluorescence was measured using a BD LSRFortessa Sorp cell analyzer (BD Biosciences, Germany).

### Peptide analysis using mass spectrometry.

Bacterial cultures were prepared as described above. A total of 1.5 ml of the diluted culture was seeded per well into 12-well culture plates with untreated surfaces (CellStar 12-well plates; Greiner Bio-One, Germany). The planktonic culture was carefully removed, 500 μl PBS was added to the well, and attached cells were removed from the surface by pipetting and scratching using a 1-ml pipette tip. The obtained suspension was centrifuged for 5 min at 4,500 × *g*. Cells were washed with the same amount of PBS and then lysed by incubation with 100 μl of a 2% sodium lauroyl sarcosinate (SLS) solution at 95°C for 15 min and subsequent sonication (Vial Tweeter; Hielscher, Germany). Cell lysates were then reduced by the addition of 5 mM Tris(2-caboxyethyl)phosphine and incubation at 95°C for 15 min, followed by alkylation (10 mM iodoacetamide for 30 min at 25°C). The cell lysates were cleared by centrifugation, and the total protein was estimated for each sample with a Pierce bicinchoninic acid (BCA) protein assay kit (Thermo Fisher Scientific, Germany). The cell lysate containing 50 μg total protein was then digested with 1 μg trypsin (Promega) overnight at 30°C in a solution containing 2% SLS and 100 mM ammonium bicarbonate for each sample. Next, SLS was removed by precipitation with 1.5% trifluoroacetic acid (TFA) and centrifugation. Peptides were purified using C_18_ microspin columns according to the manufacturer’s instructions (Harvard Apparatus, USA).

Purified peptides were dried, resuspended in 0.1% TFA, and analyzed by liquid chromatography-mass spectrometry (MS) carried out on a Q-Exactive Plus instrument connected to an Ultimate 3000 rapid-separation liquid chromatography (RSLC) nano instrument with a Prowflow upgrade and a nanospray flex ion source (all Thermo Scientific, Germany). Peptide separation was performed on a reverse-phase high-performance liquid chromatography (HPLC) column (75 μm by 42 cm) packed in-house with C_18_ resin (2.4 μm; Dr. Maisch GmbH, Germany). The following separating gradient was used: 98% solvent A (0.15% formic acid) and 2% solvent B (99.85% acetonitrile, 0.15% formic acid) to 25% solvent B over 105 min and to 35% solvent B for an additional 35 min at a flow rate of 300 nl/min. The data acquisition mode was set to obtain one high-resolution MS scan at a resolution of 70,000, with full width at half-maximum (at *m/z* 200), followed by tandem MS (MS/MS) scans of the 10 most intense ions. To increase the efficiency of MS/MS attempts, the charged-state screening modus was enabled to exclude unassigned and singly charged ions. The dynamic-exclusion duration was set to 30 s. The ion accumulation times were set to 50 ms for MS and 50 ms at a resolution of 17,500 for MS/MS. The automatic gain controls were set to 3 × 10^6^ for MS survey scans and 1 × 10^5^ for MS/MS scans. Label-free quantification (LFQ) of the data was performed as described previously (54, 55). In short, for LFQ, the raw data were uploaded to Progenesis (version 2.0; Nonlinear Dynamics), and exported .mgf files were searched by using MASCOT (version 2.5; Matrix Science). Progenesis peptide measurement exports were then further evaluated using SafeQuant for false-discovery adjustment and quality control. All experiments were performed in duplicates.

### Antibiotic sensitivity of UPEC strains.

The sensitivity of E. coli strains to antibiotics was tested on LB agar plates supplemented with antibiotics at the concentrations indicated in Table S1 in the supplemental material. Five microliters of a culture of each strain grown overnight was plated onto the respective plate and incubated at 30°C for 24 h. Antibiotic sensitivity was determined as the absence of visible colony growth.

### Data evaluation.

All experiments were done in triplicate. Differences between groups were calculated using Mann-Whitney tests. Flow cytometry data were analyzed with FlowJo v.10 software (TreeStar, USA). Proteomics data were analyzed with Perseus v.1.5.2.6. software using Student’s *t* test (56). Graphs were drawn with R v.3.6.0 software (57) using the ggplot2 v.3.2.1. package (58).

### Data availability.

The mass spectrometry proteomics data have been deposited at the ProteomeXchange Consortium via the PRIDE (59) partner repository with the data set identifier PXD020710 (https://www.ebi.ac.uk/pride/archive/projects/PXD020710).

## Supplementary Material

Supplemental file 1

Supplemental file 2

Supplemental file 3
