# Supplementary material for: Multiple Drug-Induced Stress Responses Inhibit Formation of Escherichia coli Biofilms
Source: Appl Environ Microbiol. 2020 Oct 15;86(21):e01113-20. doi: 10.1128/AEM.01113-20 (PMC7580552; doi:10.1128/AEM.01113-20)
Supplement: Supplemental file 1 [file AEM.01113-20-s0001.pdf]

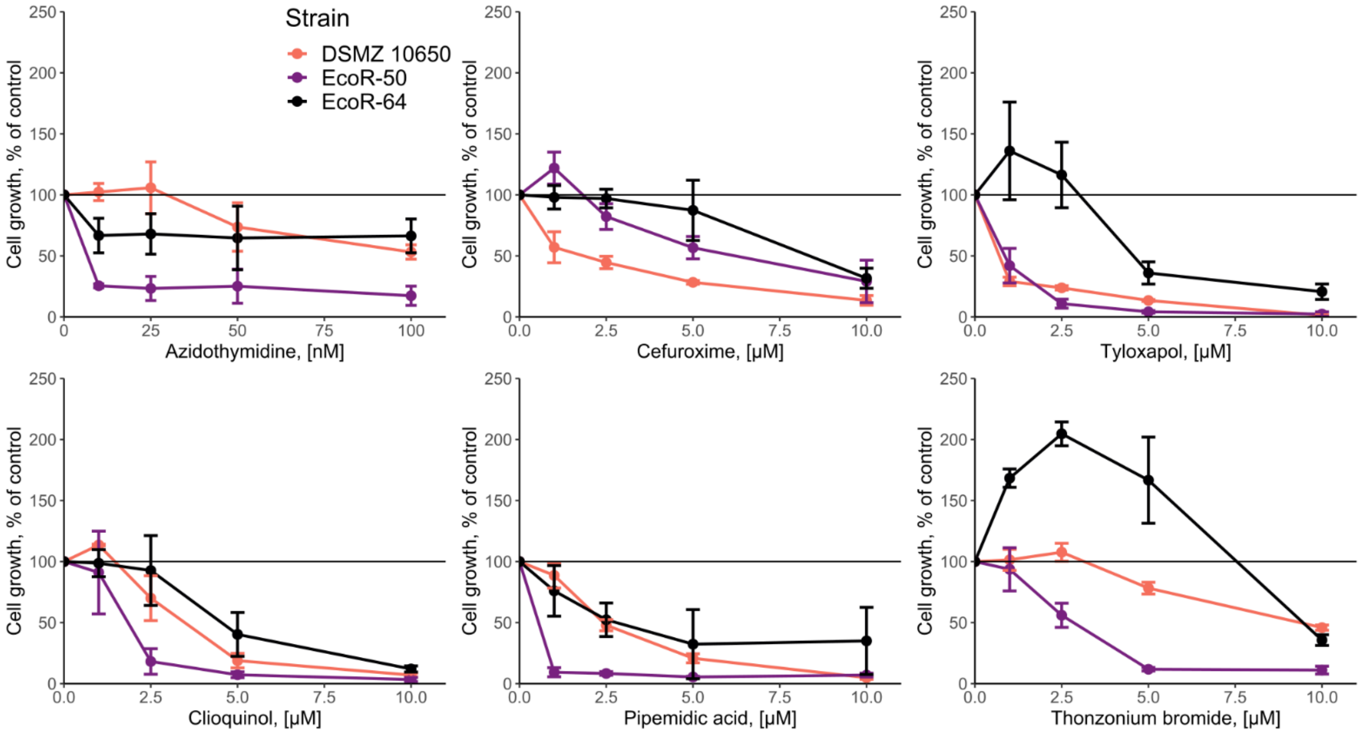

FIG S1. Dose-dependent effects of indicated drugs on biofilm formation by UPEC strains.

Cell growth in biofilms was quantified using crystal violet (CV) staining (see Materials and Methods) and values were normalized to the respective untreated strain control. All experiments were performed in triplicates. Error bars indicate standard errors.

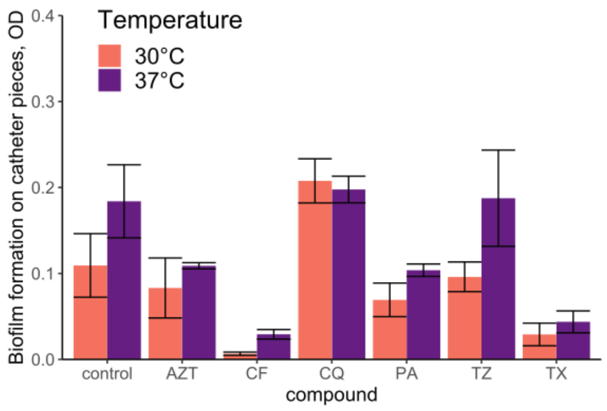

FIG S2. Effects of indicated drugs on biofilm formaiton by DMSZ 10650 on silicon urinary catheters in urine.

The cultures were grown for 48 hours at 30°C or 37°C in donor urine in presence of catheter pieces, either in absence of treatment (control) or in presence of 0.05 µm azydothimidine, 5 µm cefuroxime sodium salt, 5 µm clioquinol, 5 µm pipemidic acid, 5 µm thonzonium bromide or 2.5 µm tyloxapol, as indicated. All experiments were performed in triplicates. Error bars indicate standard errors.

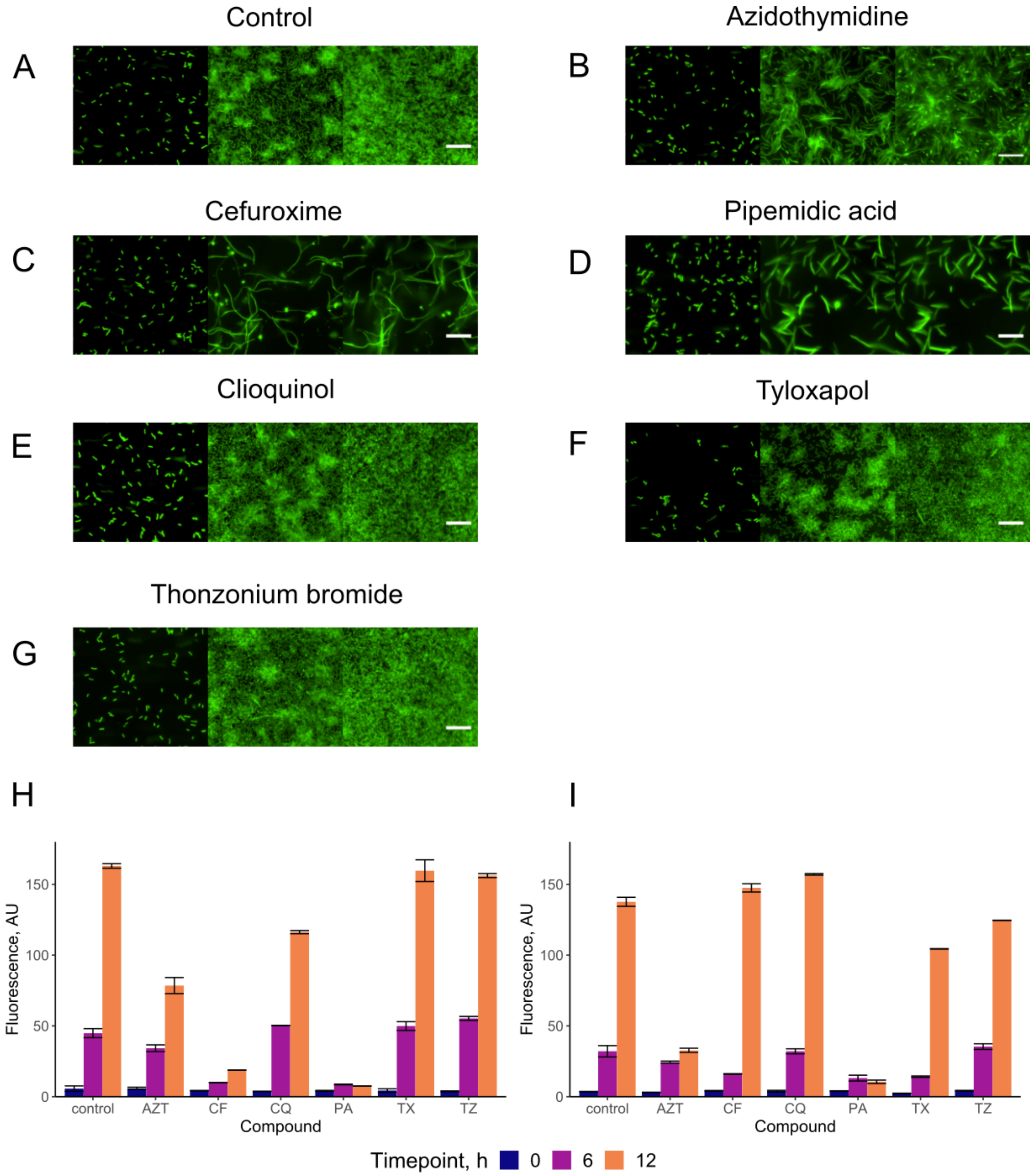

FIG S3. Early stages of biofilm formation by *E. coli* W3110 on glass surface under flow.

Representative microscopy images of cultures grown in Bioflux microfluidic chambers (see Materials and Methods) in TB medium **(A)**, or in TB medium supplemented with 0.1 µM azidothymidine **(B)**, 2.5 µM cefuroxime sodium salt **(C)**, 10 µM pipemidic acid **(D)**, 1 µM clioquinol **(E)**, 2.5 µM tyloxapol **(F)** or 5 µM thonzonium bromide **(E)**. The timepoints are 0 h, 6 h and 12 hours after the medium change to TB supplemented with respective compounds (from left to right). Exception was tyloxapol that was added already at -3 h during cell attachment. The scale bar equals 20 µm. **(H-I)** Quantification of total fluorescence in microscopy images as shown in (A-G), at same concentrations (H) or with 0.05 µM azidothymidine, 1.25 µM cefuroxime sodium salt and 5 µM pipemidic acid (I).

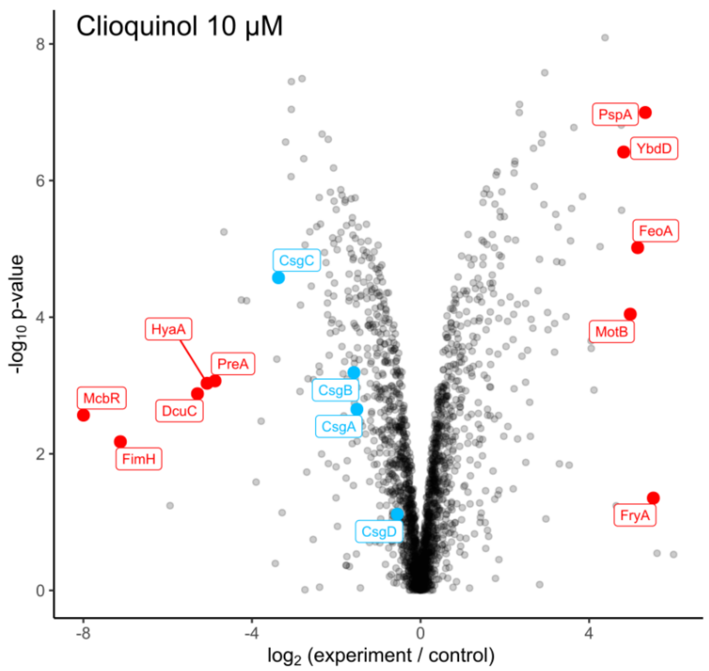

FIG S4. Global changes in protein levels for *E. coli* W3110 upon treatment with 10 µM clioquinol.

Experiments were performed and evaluated as in Figure 5.

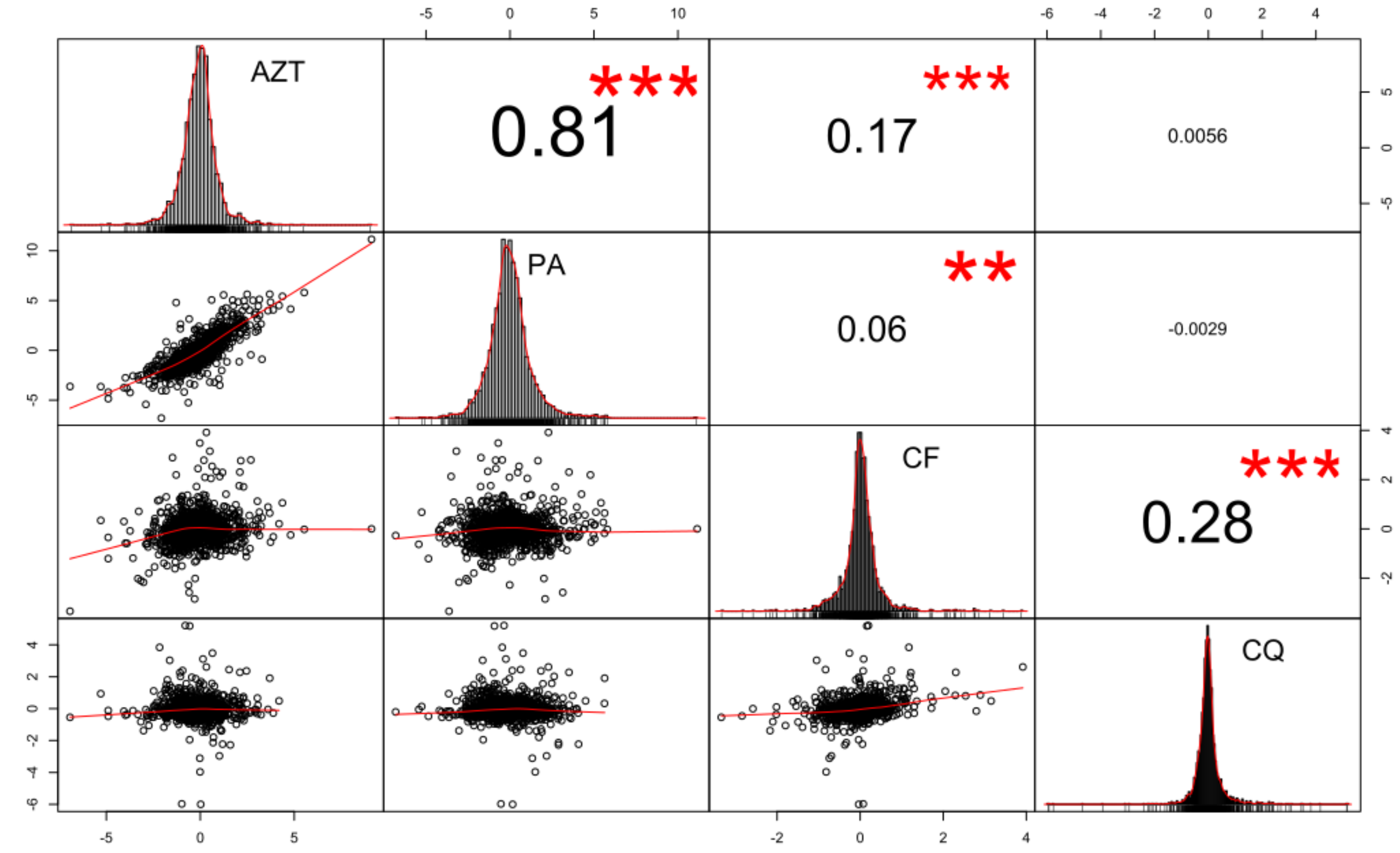

FIG S5. Correlation between proteome changes induced in *E. coli* W3110 by individual compounds.

Data are from Figure 5. The graph was drawn using chart.Correlation function within PerformanceAnalytics package in R.

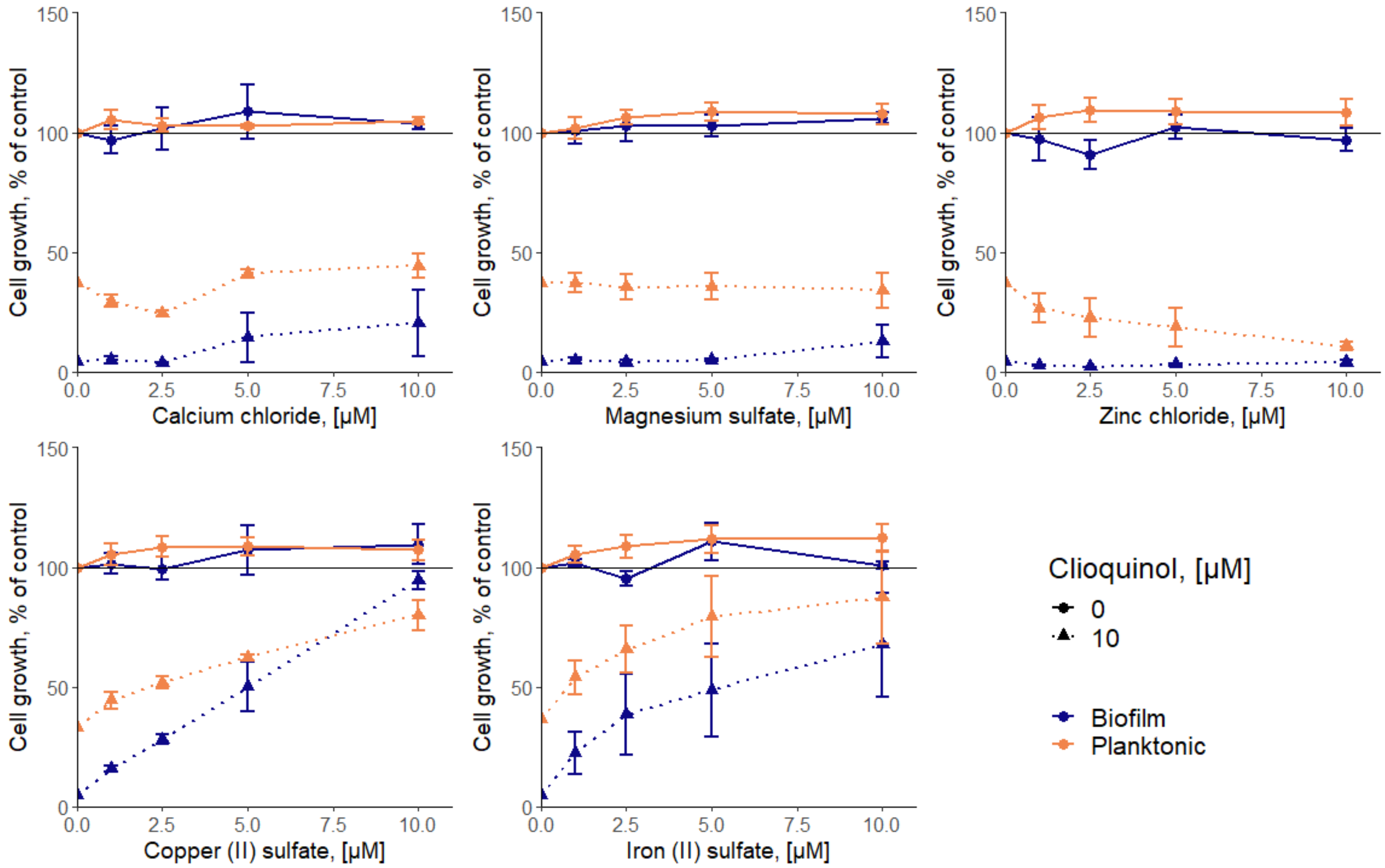

FIG S6. Dose-dependent effects of divalent cations with or without clioquinol on planktonic culture growth and biofilm formation by *E. coli* W3110.

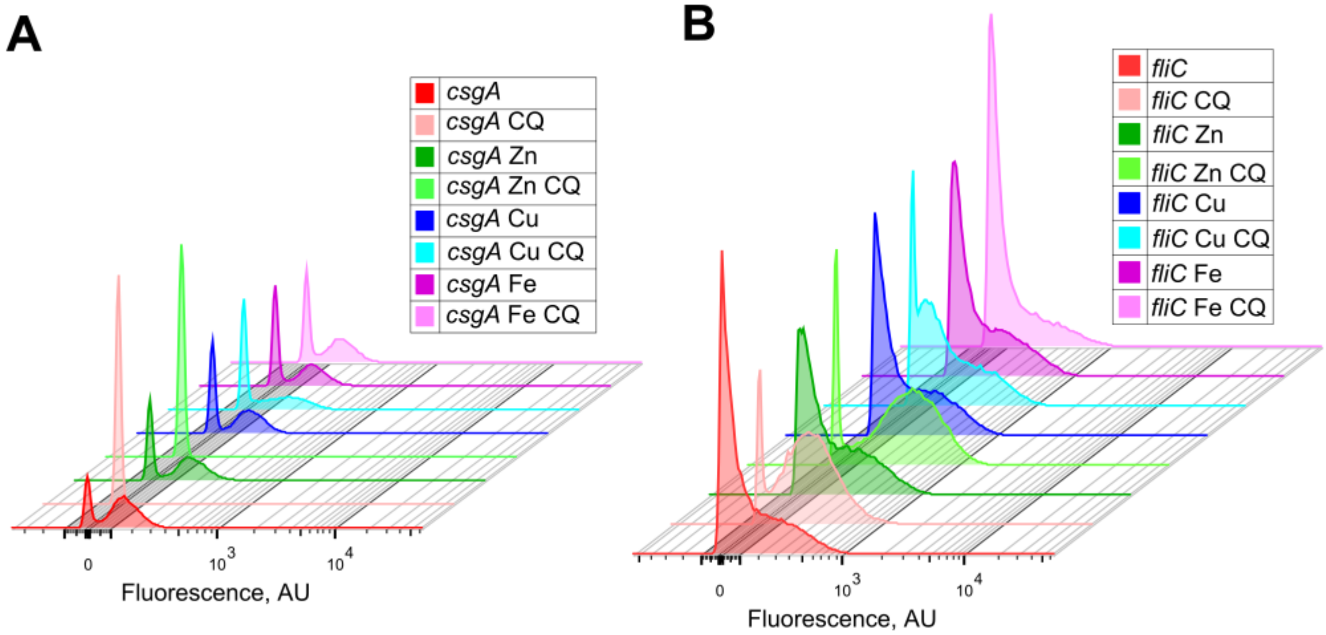

FIG S7. Effects of divalent cations on gene expression with and without clioquinol.

(A,B) Activity of *csgA* (A) and *fliC* (B) promoter reporters in cultures treated with divalent cations in absence or in presence of clioquinol. Experiments and analysis are as in Figure 6.

Table S1. Resistance of UPEC strains to some commonly used antibiotics.

	EcoR-50[a]	EcoR-64	DSMZ 10650
Ampicillin (100 µg/ml)	S	R	S
Kanamycin (50 µg/ml)	S	R	R
Chloramphenicol (34 µg/ml)	S	S	S
Tetracycline (5 µg/ml)	S	R	S
Streptomycin (100 µg/ml)	S	R	R
Gentamycin (20 µg/ml)	R	R	S

[a]Sensitivity (S) or resistance (R) of strains to antibiotics was determined by absence or presence of colony growth on LB agar plates as described in Materials and Methods.
